# Explorative Field Study on the Use of Oral Fluids for the Surveillance of *Actinobacillus pleuropneumoniae* Infections in Fattening Farms by an Apx-Real-Time PCR

**DOI:** 10.3390/vetsci9100552

**Published:** 2022-10-08

**Authors:** Michael Kleinmans, Kerstin Fiebig, Robert Tabeling, Hanny Swam, Annelies Duivelshof-Crienen, Mathias Ritzmann, Matthias Eddicks

**Affiliations:** 1 Veterinary Practice “Am Kapellhof”, 47608 Geldern, Germany; 2 Intervet Deutschland GmbH, MSD Animal Health, 85716 Unterschleissheim, Germany; 3 Intervet International B.V., 5831 AK Boxmeer, The Netherlands; 4 Clinic for Swine at the Centre for Clinical Veterinary Medicine, Ludwig-Maximilians-Universität München, 85764 Oberschleissheim, Germany

**Keywords:** surveillance, transmission, fattening

## Abstract

**Simple Summary:**

Oral fluid sampling (OFS) is an animal friendly and easy way for surveillance purposes in domestic swine populations, especially concerning respiratory diseases. In case of *Actinobacillus* (*A.*) *pleuropneumoniae* surveillance, measures are usually combined with burdensome sampling for animals and humans. In the present study, we evaluated the suitability of oral fluids (OFs) for surveillance purposes of *A. pleuropneumoniae* infections in fattening pigs using an Apx-toxin real-time PCR. We were able to demonstrate that the examination of OFs by an Apx-toxin real-time PCR is suitable for *A. pleuropneumoniae* surveillance in the field in an animal friendly and easy way. These results might contribute to an increased compliance of laboratory diagnostic measures on pig farms and thereby to increased animal welfare due to less burdensome sampling and improved animal health.

**Abstract:**

Oral fluids (OFs) represent a cost effective and reliable tool for surveillance purposes, mostly regarding viruses. In the present study, we evaluated the suitability of OFs for surveillance purposes concerning *Actinobacillus* (*A.*) *pleuropneumoniae* infections in fattening pigs under field conditions. OFs were examined with an Apx-toxin real-time PCR that detects the genes encoding for Apx I-, Apx III-, and Apx IV-toxin. For this purpose, we conducted a pen-wise collection of OFs over one fattening period from fattening pigs of two farms (farm A and B) with a known history of *A. pleuropneumoniae* infection. Lung lesions were determined at slaughter to estimate the extend of pulmonary lesions and pleural affection. Apx III- and Apx IV-toxin DNA were present in the OFs of both farms whereas Apx I-toxin DNA was present on farm A only. We were able to detect Apx I-, Apx III-, and Apx IV-toxin DNA in different patterns directly after introduction of the new pigs in the farms and over the entire study period. In summary, or results indicate the suitability of OFS for the early detection and surveillance of *A. pleuropneumoniae* in fattening farms.

## 1. Introduction

Oral fluids (OFs) are mostly used for the surveillance of viral pathogens associated with respiratory disease [1] in an efficient and cost effective way [2]. The suitability for surveillance purposes was shown for porcine reproductive and respiratory syndrome virus (PRRSV) [3,4], Influenza A virus (IAV) [1,5] or porcine circovirus type 2 (PCV2) [6,7]. Even for epidemics such as classical swine fever [8] or African swine fever [9], OFs can find a use for surveillance purposes. Concerning bacterial pathogens, in terms of *Mycoplasma* (*M.*) *hyopneumoniae,* the sensitivity under experimental conditions seems to be low [10]. To gain sufficient information from oral fluid sampling (OFS) concerning *M. hyopneumoniae* infections under field conditions, several pens at different times of rearing or fattening should be examined [1,11]. Concerning the detection of *Actinobacillus* (*A.*) *pleuropneumoniae* in OFs, DNA was detectable by a PCR targeting a gene sequence of the outer membrane lipoprotein A (omlA) under field conditions [12].

For the proof of the species *A. pleuropneumoniae*, the detection of the species-specific *A. pleuropneumoniae* repeats in toxin (Apx) IV gen by PCR is suitable [12]. The detection of Apx I–III-toxin is of particular interest, since the expression of Apx-toxin is the crucial factor to estimate the virulence of the different *A. pleuropneumoniae* serotypes (ST) [13]. The differentiation of serotypes can be achieved using a multiplex PCR system (mPCR) that targets the gene sequences of Apx I–III [14], Apx I–III in combination with omlA protein [15], and capsular polysaccharides (cps) [16] alone or in combination with omlA protein [17]. Furthermore, the continuous advancement of *A. pleuropneumoniae* mPCRs within the last years enables the differentiation of the *A. pleuropneumoniae* ST 1–19 [18,19]. In addition, a highly sensitive and specific DNA microarray has been developed that allows rapid identification and typing of previously untypable serotypes [20].

Concerning live animals, materials for the mentioned *A. pleuropneumoniae* PCRs are usually collected by invasive methods such as nasal swabbing [21], tracheobronchial lavage fluid sampling [22], or tonsil scratches [23]. These kinds of sampling are burdensome for veterinarians and animals [24]. Thus, the ease of use of OFs might possibly increase the compliance of veterinarians and farmers and thereby increase diagnostic information on farm level [25]. The present study was set up to evaluate the suitability of OFs for the surveillance of *A. pleuropneumoniae* infection based on a pen-wise collection on two fattening farms using an Apx-toxin-PCR that detects the genes encoding for ApxI-, Apx III-, and Apx IV-toxin.

## 2. Materials and Methods

### 2.1. Pigs/Farms

The study was conducted in two fattening farms located in North-Western Germany with a known history of *A. pleuropneumoniae* infection. The *A. pleuropneumoniae* status was determined by the herd attending veterinarians during routine diagnostic investigations. Further characterization of *A. pleuropneumoniae* was carried out using an Apx-toxin multiplex PCR according to Sthitmatee et al. [26] (farms A and B). In addition, it was necessary to use a multiplex PCR that targets the gene sequence of cps and omlA [17] to allow a further differentiation of *A. pleuropneumoniae* on farm B. On farm A, *A. pleuropneumoniae* ST 1/9/11 and on farm B, *A. pleuropneumoniae* ST 7 were identified. Furthermore, on farm B, *A. pleuropneumoniae* ST 3/6/8 was detected during routine diagnostics by a serotyping *A. pleuropneumoniae* ELISA (ID Screen^®^ APP Screening Indirect (serotypes 1 through 12); ID.VET; Grabels; France). Additionally, based on occasion-related diagnostics on the corresponding piglet producing farms and the fattening farms itself, both farms were stated positive for PRRSV and *M. hyopneumoniae.*

On both farms, the pigs were kept in accordance with the guidelines of the German Animal Welfare Ordinance [27]. The pigs were housed on concrete slatted floors; water was available ad libitum via nipple drinkers. The animals on both farms received a commercial diet via a sensor-controlled liquid feeder in a long trough. A negative pressure ventilation system was used on both farms. The supply air system on farm A was a slotted aisle ventilation with ceiling elements. On farm B, a door aisle ventilation system with a perforated plate door was used. Both farms managed their stable in an all-in/all-out process. Within the routine management of the farms the pigs of farm A and farm B were vaccinated against *M. hyopneumoniae* and porcine circovirus type 2 (PCV2) (Porcilis^®^ PCV M Hyo; Intervet Deutschland GmbH; Unterschleissheim; Germany) on the 3rd day of life and the 21st day of life. In addition, piglets were vaccinated against PRRSV (Suvaxyn^®^ PRRS MLV; Zoetis Deutschland GmbH; Berlin, Germany) on the 3rd day of life. Piglets on farm A were additionally vaccinated against *A. pleuropneumoniae* (Porcilis^®^ APP; Intervet Deutschland GmbH; Unterschleissheim; Germany) in the 9th and in the 13th week of life.

Farm A housed 3360 fattening pigs allocated into 12 barns (280 pigs/barn). Within the barns eight pens with 35 animals each were present. Four weeks after placement the 12 largest pigs from each pen were sorted out and moved to another compartment (Table 1).

On farm A, 280 pigs with an average live weight of 21.2 kg and age of nine weeks were included in the examination. All pigs were housed within the same barn. From study week four (W4) on, 23 animals per pen were still integrated in the study. These groups remained stable until slaughter.

Farm B housed 1080 fattening pigs in five different barns. The entire farm is managed all-in/all-out. In total 216 pigs of an entire barn with an average live weight of 30.9 kg and an age of eleven weeks, which were evenly distributed among eight pens (27 animals per pen), were included in the examination. Until slaughter, no changes were made to the group composition.

Antimicrobial treatment was carried out using single parenteral treatment within the routine herd health management of the herd attending veterinarians, if necessary (detailed information in the Supplementary Materials).

### 2.2. Data Collection

#### 2.2.1. Sampling of OFs

For the collection of OFs, cotton ropes (Oral Fluid Sample Collection Accessory Kit; IDEXX^®^ Laboratories Inc., Westbrook, ME, USA) were used. The sampling period lasted 25 minutes as described by Prickett et al. [28]. One rope was used for approximately 25 animals [1]. Within the first week after placement, the sampling was conducted on a daily basis (D0–D7). Afterwards, OF samples were collected every two weeks (week 3, 5, 7, 9, 11, 13, 15, 17, and 19). The number of OFs per pen varied according to the number of animals (one OFs per 25 animals) (Table 1). On farm A, additional OFs were obtained in W4, on the day of the second *A. pleuropneumoniae* vaccination. On the two vaccination dates (D0 and W4), the OFs were collected before vaccination.

The ropes were placed in an area with distance to nipple drinkers, feeding troughs, and neighboring pens to avoid contaminations. Whenever OFs were collected, the position of the cotton ropes was adjusted to the size of the growing animals in such a way that the lower end of each rope was at the pigs’ shoulder height [1,28]. After the exposure phase, the OFs were extracted by wringing out the soaked ropes in plastic bags. An integrated, sealable 5 mL collection container (Oral Fluid Sample Collection Accessory Kit; IDEXX^®^ Laboratories Inc., Westbrook, ME, USA) was placed at the bottom of the plastic bag. The collection container was then removed and sealed. To prevent cross-contamination, new disposable gloves were worn for each OF.

#### 2.2.2. Evaluation of Lung Lesions

Pleural lesions were assessed using the Slaughter Pleuritis Evaluation System (SPES score) [29]. This assesses the prevalence, extent, and location of pleurisy with a score ranging from zero to four. Enzootic pneumonia (EP)-type lung lesions were assessed using an already published method [30].

#### 2.2.3. Molecular Biological Examinations of OFs

The OFs were examined by a commercially available *A. pleuropneumoniae* real-time PCR (qPCR) (*Actinobacillus pleuropneumiae* qPCR test kit; BioCheck UK Ltd., Ascot, UK) in the Center for Diagnostic Solutions of MSD Animal Health in the Netherlands. This PCR is able to quantify the amount of Apx DNA targets in a sample [31] and is validated by BioCheck UK Ltd. for the investigation of OFs. The used primers of the multiplex qPCR are specific for Apx IV (FAM channel), Apx I (Cy 5 channel), and Apx III (Texas Red channel). The respective amount of DNA detected is expressed in logarithm (log)10 copies (c) per microliter (µL) (log10 c/µL).

### 2.3. Statistical Analysis

For the statistical analysis the program IBM SPSS Statistics^®^ (version 26.0, IBM^®^ SPSS Inc., Chicago, IL, USA) and Microsoft EXCEL^®^ (version 2016, Microsoft Office, Redmond, WA, USA) were used. Metric data were tested for normal distribution by Kolmogorov-Smirnov tests. In case of normal distribution Student’s *t*-Test was used to compare groups. In case of non-normally distributed data, statistical analysis was performed using Mann-Whitney-U test or Spearman-rho correlation. Values *p* < 0.05 were considered statistically significant. The confidence interval was 0.95.

## 3. Results

On farm A, one animal died nine weeks after placement and on farm B one pig died seven days after placement in the fattening units. Thus, the mortality in the study-population was 0.54% on farm A and 0.46% on farm B. In both cases, a postmortem examination at an accredited pathological examination service (farm A: State Laboratory Rhein-Ruhr-Wupper (CVUA-RRW), Germany; farm B: State Laboratory of Emscher-Lippe (CVUA Emscher-Lippe), Germany was carried out. The examination of the pig from farm A revealed a catarrhal-suppurative pneumonia with unspecific bacterial pathogen detection. The pig from farm B showed chronic-active suppurative lung lesions with abscess formation and focal pleuritis. The bacteriological examination revealed the detection of *Streptococcus suis* in those lesions.

**Figure 1:**
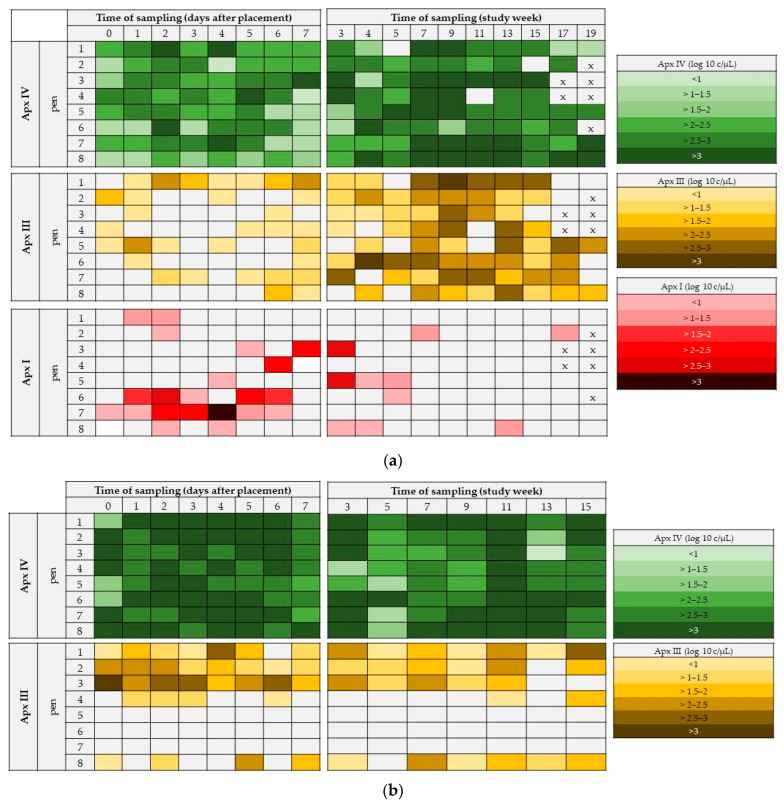
(**a**) Farm A: Apx I-, Apx III-, and Apx I-toxin DNA positive pens within the first week after placement and the entire study period. Different colored squares indicate different DNA-loads in the OFs. All pigs on farm A were vaccinated against *A. pleuropneumoniae* (Porcilis^®^ APP; Intervet Deutschland GmbH; Unterschleissheim; Germany) at day 0 after placement and 4 weeks after placement. (**b**) Farm B: Apx IV-, and Apx III-toxin DNA positive pens within the first week after placement and the entire study period. Different colored squares indicate different DNA-loads in the OFs. X = no samples collected.

Overall, 447 OFs (217 from farm A and 230 OFs from farm B) were available for the molecular biological examinations. In total, 94.2% (95% CI: 91.7–96.2%) of all OFs were positive for Apx IV-toxin DNA (farm A: 91.7%; 95% CI: 89.0–94.9%; farm B: 96.5%; 95% CI: 93.9–98.7%). Apx III-toxin DNA in OFs was detected in 43.4% (95% CI: 39.1–47.9%) of all OFs (farm A: 48.8%; 95% CI: 41.9–55.3%; farm B: 38.7%; 95 % CI: 33.0–45.2%) and Apx I-toxin DNA was present in 9.4% (95 % CI: 6.7–12.1%) of all OFs, but positive ones originated all from farm A (19.4%; 95% CI: 13.8–24.9%). Apx IV-toxin DNA was continuously detected in all pens over the entire study period except on farm A when three pens were negative for Apx IV-toxin DNA at one occasion of sampling, whereas Apx IV-toxin DNA was present in all OFs and at all times of samplings of farm B. Concerning the detection of Apx III-toxin DNA, each pen was Apx III-toxin DNA positive at least once within the sampling period on farm A, whereas three pens on farm B remained negative for Apx III-toxin DNA in OFs over the entire study period. Apx I-toxin DNA was detected at least once in OFs within the sampling period in each pen of farm A, but not on farm B. An overview on the detection and semiquantitative evaluation of Apx IV-, Apx III-, and Apx I-toxin DNA at different times of sampling under respect of each pen is presented in Figure 1a,b for farm A and farm B, respectively.

### 3.1. OFs on Pen-Level at Different Times of Sampling

For the detailed pattern of pen wise detection please refer to Figure 1a,b. To evaluate a possible effect of the stress in parallel to the regrouping after placement in the fattening unit, we examined the OFs within the first week on a daily basis. A significant association between day of sampling and the quantitative PCR outcome was present for Apx IV-toxin DNA in the OFs on both farms. Shortly, on farm A, Apx IV-toxin DNA loads were significantly higher on study days 2 and 5 compared to day 0 directly after placement (0–2: *p* = 0.029; 0–5 *p* = 0.039) and significantly lower on study day 7 compared to study day 2 (*p* = 0.044). On farm B, Apx IV-toxin DNA loads were significantly higher on study days 0, 2, and 5 compared to study day 7 (0–7: *p* = 0.008; 2–7: *p* = 0.022; 5–7: *p* = 0.001).

**Figure 2:**
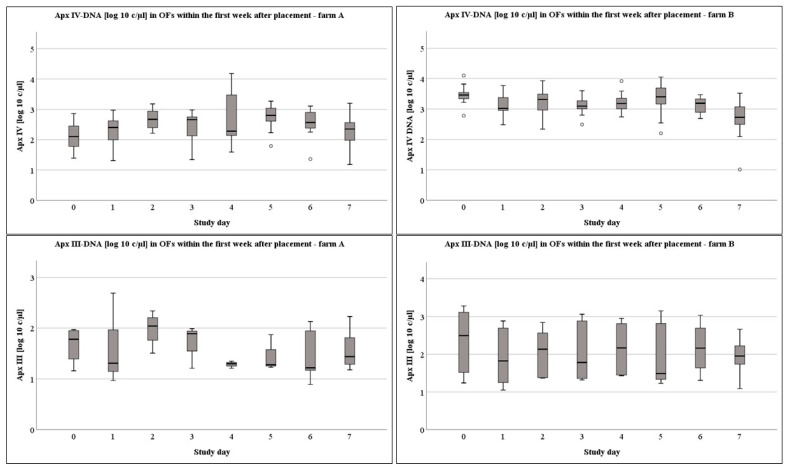
Boxplots of log10 c/µL Apx IV- and Apx III-toxin DNA in OFs from placement until 7 days after placement from farms A and B.

**Figure 3:**
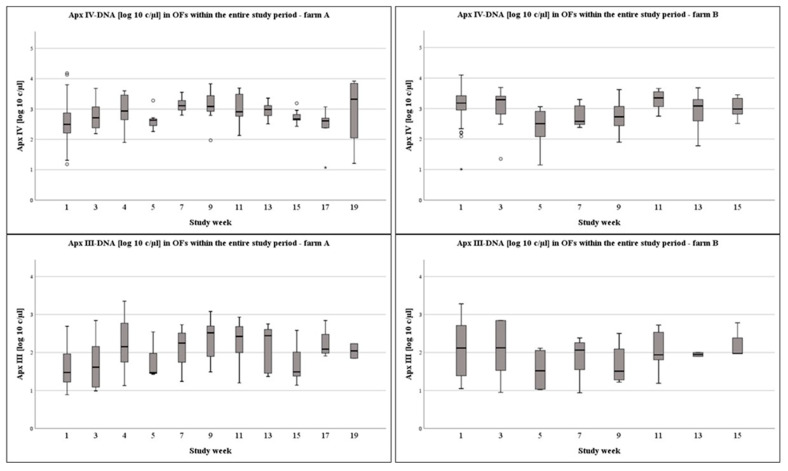
Boxplots of log10 c/µL Apx IV- and Apx III-toxin DNA in OFs over the entire study period at a weekly basis from farms A and B.

**Figure 4:**
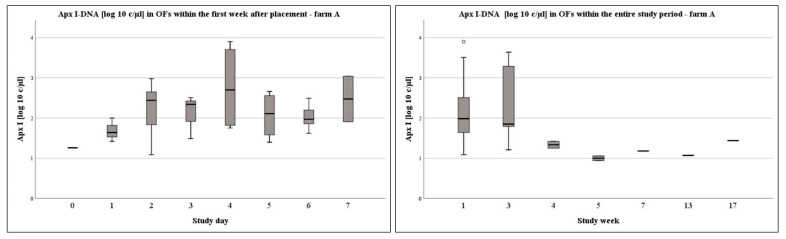
Boxplots of log10 c/µL Apx I-toxin DNA load in OFs of farm A within study week one and over the entire study period at a weekly basis.

On a weekly basis, on farm A, Apx IV-toxin DNA loads were significantly higher in week 7 compared to the first week of sampling (*p* = 0.014). On farm B, Apx IV-toxin DNA loads in study week 1 (*p* < 0.001), week 3 (*p* = 0.014), and week 11 (*p* < 0.001) were significantly higher compared to week 5. Furthermore, Apx IV-toxin DNA loads were significantly higher in OFs at week 1 and week 11 compared to weeks 7 or week 9 (1–7: *p* = 0.005; 1–9: *p* = 0.044; 11–7: *p* = 0.005; 11–9: *p* = 0.023). The DNA-loads in the OFs at different times of sampling are presented in Figure 2, Figure 3 and Figure 4 for the Apx I-, Apx III-, and Apx IV-toxin DNA. Please consider that Apx I-toxin DNA was only present on farm A.

The overall correlation (Spearman’s rho) between the quantitative outcome of the PCR concerning Apx IV and Apx III or Apx I was in a moderate but on a significant level (farm A: Apx IV-Apx III: r_s_ 0.355, *p* < 0.001; Apx IV-Apx I: r_s_ 0.218, *p* = 0.001; farm B: Apx IV-Apx III: r_s_ 0.340, *p* < 0.001). Concerning different times of sampling, correlations were present in an irregular pattern (Table 2).

### 3.2. Lung Lesion Scoring at Slaughter

At the end of the study period, we were able to assess the lung lesions of 327 pigs in total (149 lungs of pigs from farm A and 178 lungs of pigs from farm B). In total, 64.4% (95% CI: 59.4–69.3%) of all lungs showed EP-type lesions (farm A: 62.8%; 95% CI: 54.7–70.3%; farm B: 65.7%; 95% CI: 58.3–72.6%) with a mean score of 3.41 ± 2.56 (farm A: 3.52 ± 2.93; farm B: 3.33 ± 2.21). Pleuritis was present in 7.0% (95% CI: 4.6–10.1%) of the lungs from pigs of both farms (farm A and 6.7%; 95% CI: 2.7–11.4%; farm B 7.3%; 95% CI: 3.9–11.2%). In total, 60.9% (95% CI: 38.9–81.8%) of the cases were defined as cranial pleuritis (farm A: 60.0%; 95% CI: 25–91.7%; farm B: 61.5%; 95% CI: 31.3–88.9%) and 39.1% (95% CI: 18.2–61.2%) of the cases were categorized as caudo-dorsal pleuritis (farm A: 40.0%; 95% CI: 8.3–75%; farm B: 38.5%; 95% CI: 11.1–68.7%).

## 4. Discussion

Surveillance of *A. pleuropneumoniae* infection in live animals can be carried out by the screening for antibodies either against *A. pleuropneumoniae* antigen [32] or Apx-toxins in serum samples [33,34] as well as by the detection of *A. pleuropneumoniae* DNA by PCR and bacteriological examinations of tonsil scratches [21]. However, both approaches are time consuming and burdensome for the animals and veterinarians [24]. Farms with a high health status and unsuspicious regarding *A. pleuropneumoniae* should ensure that this status is maintained not only for health but also for economic reasons [35,36]. Monitoring programs as ongoing test programs with the intention to detect infections at an early stage of production are the means of choice [35]. Concerning *A. pleuropneumoniae* surveillance based on the detection of antibodies, a lack of seroconversion due to an infection restricted to the tonsils can lead to incorrect interpretation of the results [37]. Thus, the use of tonsil scratch samples is currently considered as suitable for the early recognition of subclinical infected pigs [23]. The correct sample size selection is also recognized as a critical factor in the successful implementation of screening and monitoring programs to avoid false negative results. In relation to this, the expected prevalence of *A. pleuropneumoniae* infections is of particular importance when groups of animals are examined [38]. The monitoring programs commonly used so far are time and cost intensive, and in addition, they are burdensome for the animals and farmers as well [3,24,25]. The approach using OFs for surveillance purposes enables a fast, non-invasive, and animal-friendly collection of sampling material with a large range of sampled animals [1,3,24]. Our examination was designed to longitudinally evaluate the *A. pleuropneumoniae* status of two fattening farms by pen-wise collection of samples by avoiding invasive methods. Besides the gentle way of this kind of sampling for diagnostic purposes, we believe that the ease of the collection increases the compliance of farmers for diagnostics especially in case of burdensome sample procedures like tonsil scratches or blood sampling to detect *A. pleuropneumoniae* in live pigs.

On both study farms, we observed no clinical outbreaks of *A. pleuropneumoniae* despite the detection of *A. pleuropneumponiae* specific Apx IV-toxin DNA in varying combination with Apx I- and Apx III-toxin DNA. Concerning farm A, the vaccination against *A. pleuropneumoniae* most likely reduced the clinical and pathomorphological outcome of the infection, whereas we assume a subclinical infection on farm B. Furthermore, it should be noted that an outbreak of a clinical *A. pleuropneumoniae* infection is a multifactorial event, which requires further triggers that may not have been present [39]. However, the detection of Apx IV-toxin DNA in 91.7% of the examined OFs on farm A and in 96.5% of the examined OFs on farm B under these circumstances underlines the sensitivity of this method. This is of particular interest as subclinically infected animals depict a threat to *A. pleuropneumoniae* negative herds [35]. Thus, OF sampling in advance of the introduction of animals in naive pig herds might be an appropriate measure to avoid unwanted transmission of *A. pleuropneumoniae*. Within our examination, we were able to detect a high prevalence of Apx IV-toxin DNA, whereas Apx III- and Apx I-toxin DNA showed different patterns. Interestingly, all Apx-toxins that were present over the entire study period could be detected as soon as directly after placement of the pigs in the fattening units. However, patterns of detection may vary from farm to farm, and sample collection for the evaluation of the *A. pleuropneumoniae* status of farms or animals should be conducted on several days after placement.

The overall correlation between the quantitative outcome of the PCR concerning Apx IV- and Apx III- or Apx I-toxin indicates that virulent *A. pleuropneumoniae* strains were present in the farms as the replication of the bacteria and the detection of the corresponding Apx-DNA in our samples must go hand in hand and Apx-toxins are the decisive factor influencing the virulence of *A. pleuropneumoniae* serotypes [13]. However, correlations were not present at all times of sampling. Thus, other *Actinobacillus* species as *A. suis* or *A. rossii* [40] also exhibiting genes for Apx-toxin I or III, respectively, have most likely been present in the pig population. Based on the information of the herd attending veterinarians, farm A was positive for *A. pleuropneumoniae* ST 1/9/11, and farm B was categorized *A. pleuropneumoniae* ST 3/6/8 and ST 7 positive. Concerning farm A, we were able to detect species-specific Apx IV- and Apx III-toxin DNA. From that point of view, additional *A. pleuropneumoniae* STs must have been present on the farm that additionally encodes for Apx III-toxin (*A. pleuropneumoniae* STs: 2, 3, 4, 6, 8, or 15). Possibly *A. rossii* might interfere with our diagnostic approach, but the partially strong correlation between Apx IV-toxin DNA and Apx III-toxin DNA detection supports the above-mentioned hypothesis. Concerning farm B, the detection of Apx III-toxin DNA matches with the estimated *A. pleuropneumoniae* status of the farm.

Transportation and regrouping within the first week of placement is considered as a stressful event for the animals. As bacterial species can respond to changes in the stress hormone levels of their host environment [41,42] and stressful events like crowding or moving and mixing of pigs may be involved and contribute to the development and spread of the disease [43], we collected the OFs daily within the first week after placement to evaluate differences concerning the bacterial Apx-toxin load in OFs. On both farms, we were able to proof *A. pleuropneumoniae* infections by the detection of the species-specific Apx IV-toxin DNA [12] by PCR in OFs as early as the day of placement. Indeed, we recognized higher DNA loads of Apx IV-toxin DNA on both farms within the days after placement with a tendency to be higher two and five days after placement. However, no significant influence on the detection rate was obvious. Thus, stress associated with transport or regrouping might influence the quantitative PCR-outcome concerning Apx IV-toxin DNA detection and as a consequence thereof a higher bacterial load, but this did not impair the frequency of Apx IV-toxin DNA detection in OFs.

Three pens on farm B remained negative for Apx III-toxin DNA over the entire study period. Since the validation of the used Apx PCR for OFs showed a high sensitivity and specificity of more than 99% (Datapack *Actinobacillus pleuropneumoniae* DNA Test Kit), it can be assumed that the pigs in these pens remained negative for Apx III-toxin, or we just missed the times of Apx-III-toxin positivity within our study period [37]. However, the sampling interval in our study was narrow, and the sample size was high. The assumption that the three pens mentioned before remained negative for Apx III-toxin DNA over the entire study period despite there being direct Apx III-toxin DNA positive neighboring pens leads to the hypothesis that *A. pleuropneumoniae* spreads slowly between pens. This observation is in line with results of a study on the transmission of *A. pleuropneumoniae* in pigs, where the transmission rate was found to be ten times higher within a pen than between pens [44]. Although airborne transmission was observed in some studies [45,46], the presumably low transmission between the pens in our examination may be the result of a subclinical infection or rather low bacterial loads in the corresponding herds.

## 5. Conclusions

In the present study, we were able to demonstrate that OFs can be used for rapid detection and surveillance of *A. pleuropneumoniae* infections in fattening farms by an Apx-toxin PCR. Due to its high sensitivity, the use of OFs might be of additional value to avoid the introduction of subclinical infected pigs to *A. pleuropneumoniae* naive pig herds within the context of pre-examinations.

## Figures and Tables

**Table 1:** Number of collected OFs per sampling time point for each pen and total collected OFs.

	Farm A			Farm B		
Time of Sampling	Pigs/Pen	Collected OFs/Pen	Total Collected	Pigs/Pen	Collected Ofs/Pen	Total Collected
D 0–7	35	2	128	27	2	128
W 3	35	2	16	26–27	2	16
W 4	35	2	16	-	-	-
W 5–11	22–23	1	32	26–27	2	64
W 13	23	1	8	0–26	1–2 *	14
W 15	8–21	1	8	1–15	1 *	8
W17	0–8	0–1 *	6	-	-	-
W 19	0–2	0–1 *	4	-	-	-
Total			218			230

* The number of OFs per sampling depends on the number of pigs per pen. For every 25 pigs per pen, one OFs was collected.

**Table 2:** Correlations (Spearman’s rho) between the quantitative outcome of the Apx-PCR result of different Apx-toxin DNA at different times of sampling and the entire study period.

Farm	Time	Apx IV–Apx III	Apx IV–Apx I
A	total	r_s_: 0.355, *p* < 0.001	r_s_: 0.218, *p* = 0.001
	W1	-	r_s_: 0.377, *p* < 0.001
	W4	r_s_: 0.746, *p* = 0.001	-
	W5	-	r_s_: 0.764, *p* < 0.027
B	Total	r_s_: 0.340, *p* < 0.001	-
	W1	r_s_: 0.364, *p* < 0.001	-
	W15	r_s_: 0.741, *p* < 0.036	-

## Data Availability

Data are available from the correspond.

## Supplementary Materials

The following supporting information can be downloaded at: https://www.mdpi.com/article/10.3390/vetsci9100552/s1. Content S1: Detailed description of antimicrobial treatments during the entire study period on farm A and farm B. Figure S1: Percent Apx IV, Apx III, and Apx I positive OFs within the entire study period and the first week after placement on different occasions of sampling.
