# Supplementary material for: Explorative Field Study on the Use of Oral Fluids for the Surveillance of Actinobacillus pleuropneumoniae Infections in Fattening Farms by an Apx-Real-Time PCR
Source: Vet Sci. 2022 Oct 8;9(10):552. doi: 10.3390/vetsci9100552 (PMC9607612; doi:10.3390/vetsci9100552)
Supplement: Supplementary file 1 [file vetsci-09-00552-s001.zip › vetsci-1887461-supplementary.pdf]

veterinary sciences

MDPI

Article

Explorative Field Study on the Use of Oral Fluids for the Surveillance of *Actinobacillus pleuropneumoniae* Infections in Fattening Farms by an APX-Real-Time PCR

Content S1. Detailed description of antimicrobial treatments during the entire study period in farm A and farm B:.

On farm A 8/280 pigs received antimicrobial treament. 4 pigs were treated with amoxicillintrihydrate (Aulicin Amoxi LA 150 mg/ml, Dechra Veterinary Products Deutschland GmbH, Aulendorf, Germany; streptococcus infection; 10 mg/kg body wight, 3 consecutive days) and 4 animals were treated with florfenicol (Florkem 300mg/ml, CEVA Tiergesundheit GmbH, Düsseldorf, Germany; respiratory infection; 20 mg/kg body wight, twice in the distance of 48 hours).

On farm B 20/216 pigs received antimicrobial treatment. 15 pigs were treated with florfenicol (Florkem 300mg/ml, CEVA Tiergesundheit GmbH, Düsseldorf, Germany; respiratory infection; 20 mg/kg body wight, twice in the distance of 48 hours), 2 pigs were treated with amoxicillintrihydrate (Aulicin Amoxi LA 150 mg/ml, Dechra Veterinary Products Deutschland GmbH, Aulendorf, Germany; streptococcus infection; 10 mg/kg body wight, 3 consecutive days) and 2 pigs received tiamulin (Denagard pro inj. 100 mg/ml, Elanco Animal Health, Bad Homberg, Germany; Mycoplasma hyosynoviae; 20 mg/kg body wight; 3 consecutive days).

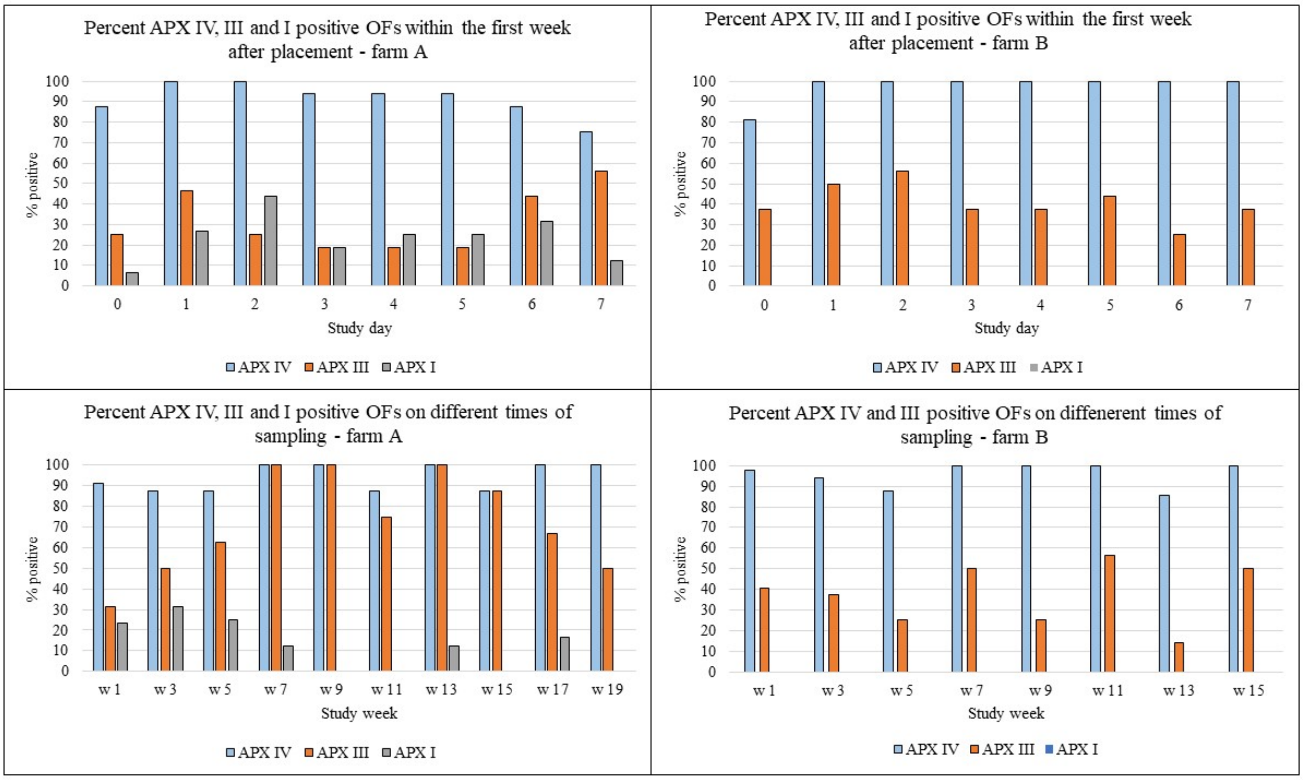

Figure S1. Percent APX IV, APX III and APX I positive OFs within the entire study period and the first week after placement on different occasions of sampling.
